# Supplementary material for: Atomic resolution cryo-EM at 200 keV
Source: IUCrJ. 2026 Jun 10;13(Pt 4):343–53. doi: 10.1107/S2052252526004100 (PMC13324606; doi:10.1107/S2052252526004100)
Supplement: Supplementary file 1 [file m-13-00343-sup1.pdf]

IUCrJ

Volume 13 (2026)

Supporting information for article:

Atomic resolution cryo-EM at 200 keV

Radostin Danev, Haruaki Yanagisawa, Keitaro Yamashita, Fabian Eisenstein and Masahide Kikkawa

Supplementary Information:

Atomic resolution cryo-EM at 200 keV

Radostin Danev[1]*, Haruaki Yanagisawa[1], Keitaro Yamashita[2], Fabian Eisenstein[3], Masahide Kikkawa[1]

[1]Graduate School of Medicine, The University of Tokyo, 7-3-1 Hongo, Bunkyo-ku, Tokyo, 113-0033, Japan

[2]Research Center for Advanced Science and Technology, The University of Tokyo, 4-6-1 Komaba, Meguro-ku, Tokyo, 153-8904, Japan

[3]DECTRIS Ltd., Taefernweg 1, 5405 Baden-Daettwil, Switzerland

Table S1. Cryo-EM data collection, refinement, and validation

	**200 kV dataset**<br>EMPIAR-13202<br>EMD-68251<br>PDB-22FX	**100 kV dataset**<br>EMPIAR-13203<br>EMD-68204
**Data collection**		
Magnification (indicated)	150,000	500,000
Voltage (kV)	200	100
Camera	Gatan K3 (CDS)	DECTRIS SINGLA
Energy filter slit (eV)	20	20
Objective aperture (μm)	-	250
Grid type	UltrAuFoil R0.6/1	UltrAuFoil R1.2/1.3
Acquisition pattern	3 x 3 x 1	3 x 3 x 4
Beam diameter (μm)	0.95	0.53
Physical pixel size (Å)	0.3056	1.17
Exposure rate (e^- pixel^{-1} s^{-1})	3.3	23.8
Exposure time (s)	1.51	3.0
Electron exposure (e^- Å^{-2})	53.4	52.2
Defocus range (μm)	-0.2 – -0.8	-0.2 – -0.8
Movies (no.)	13,654	17,424
Frames (no.)	81	13,500
Fractions (no.)	81	60
**Data processing**		
Symmetry imposed	O	O
Initial particle images (no.)	652,257	349,779
Final particle images (no.)	615,248	250,077
Map resolution, FSC 0.143 (Å)	1.24	1.91
Map resolution range (Å)	1.22 – 1.25	1.85 – 1.91
**Model Refinement**		
Initial model used	PDB-7A4M	
Model composition		
Non-hydrogen atoms	1814	
Protein residues	173	
Metals	2	
Water molecules	201	
R.m.s. deviations		
Bond lengths (Å)	0.0138	
Bond angles (°)	1.90	
Validation		
MolProbity score	1.44	
Clashscore	5.97	
Poor rotamers (%)	1.12	
CaBLAM outliers (%)	0.6	
Ramachandran plot		
Favored (%)	97.7	
Allowed (%)	2.3	
Outliers (%)	0	

Table S2. Effect of radiation damage by pre-exposure on the resolution

Omitted initial movie frames	Corresponding pre-exposure ($e^- Å^{-2}$)	3D refinement resolution (Å)	Theoretical estimate (Å)	Theoretical estimate, 82% exposure (Å)
0	0	1.66	1.66	1.66
5	3.3	1.68	1.83	1.79
10	6.6	1.81	2.03	1.95
15	9.9	1.96	2.29	2.14
20	13.1	2.15	2.60	2.37
25	16.4	2.48	2.96	2.63
30	19.7	2.83	3.35	2.93
35	23.0	3.24	3.76	3.25
40	26.3	3.58	4.17	3.58

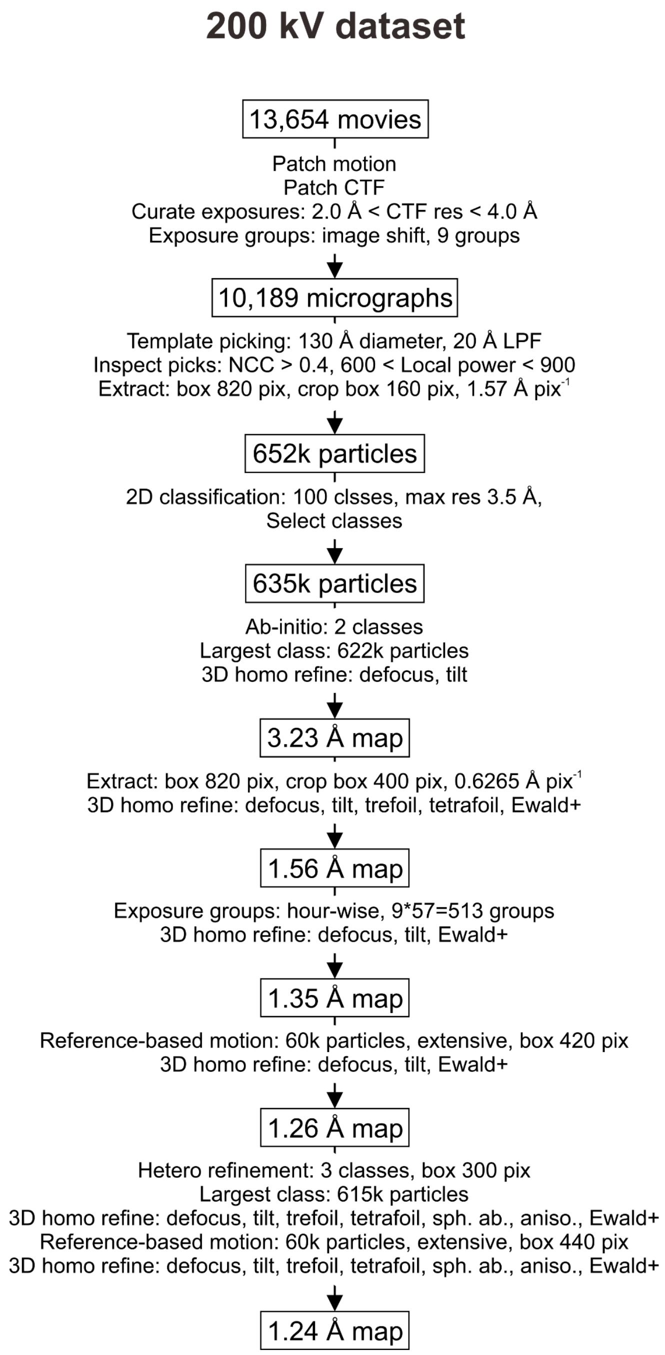

Supplementary Figure S1. Data processing workflow for the 200 kV dataset

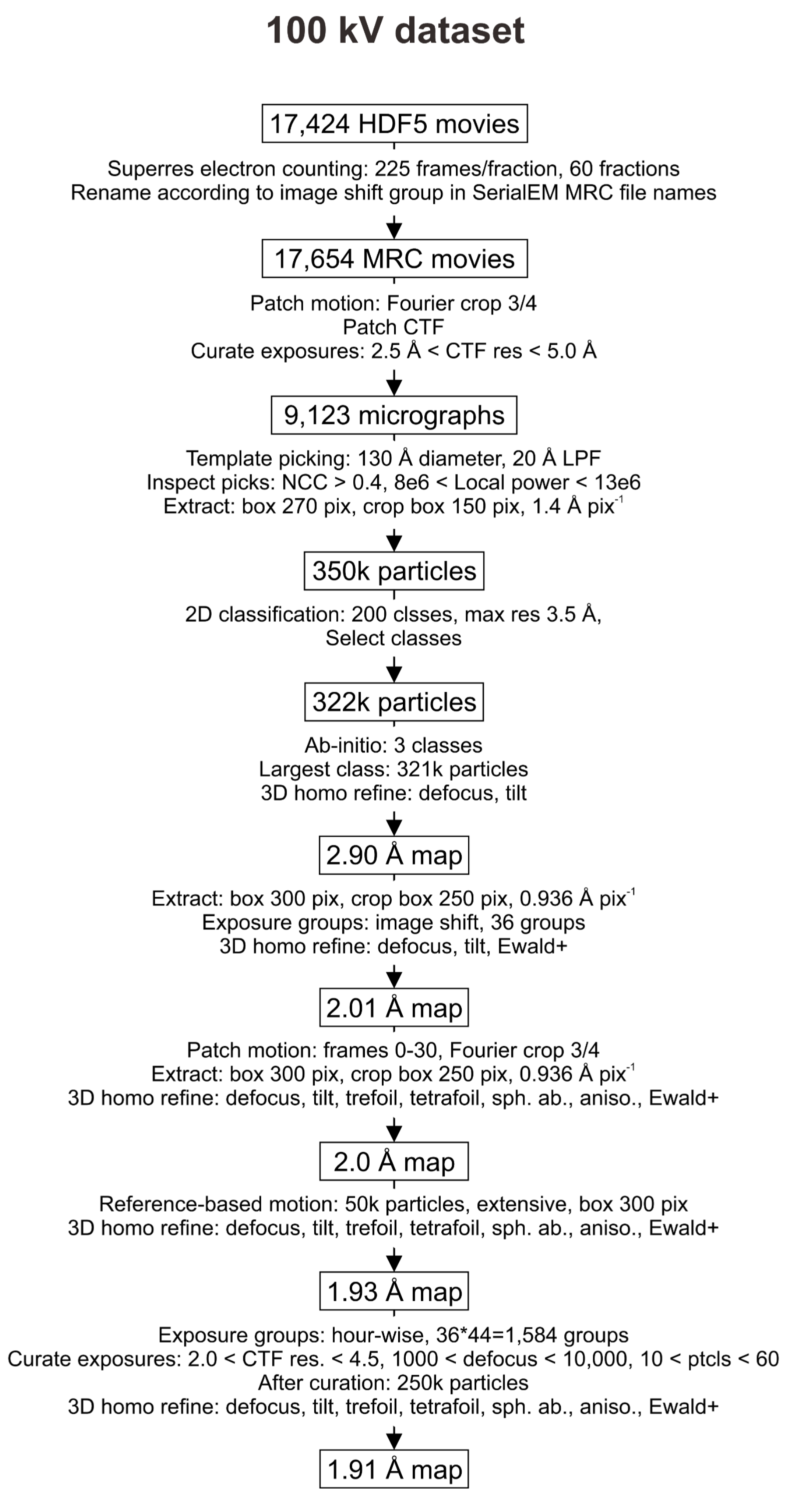

Supplementary Figure S2. Data processing workflow for the 100 kV dataset

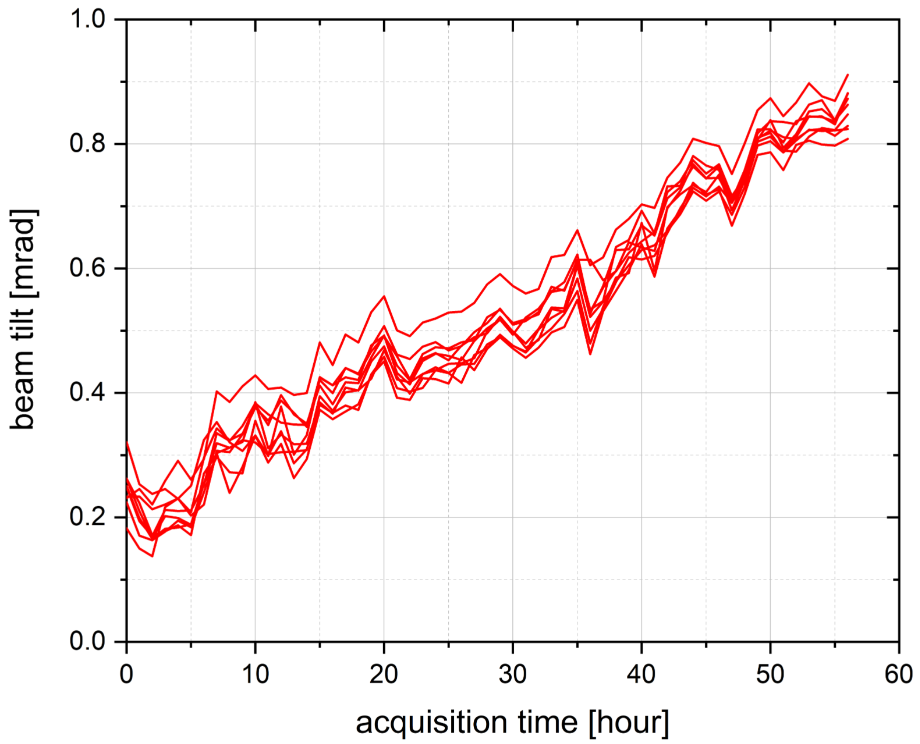

Supplementary Figure S3. Beam tilt evolution for the nine image shift exposure groups over the acquisition period of the 200 kV dataset.

(*a*)

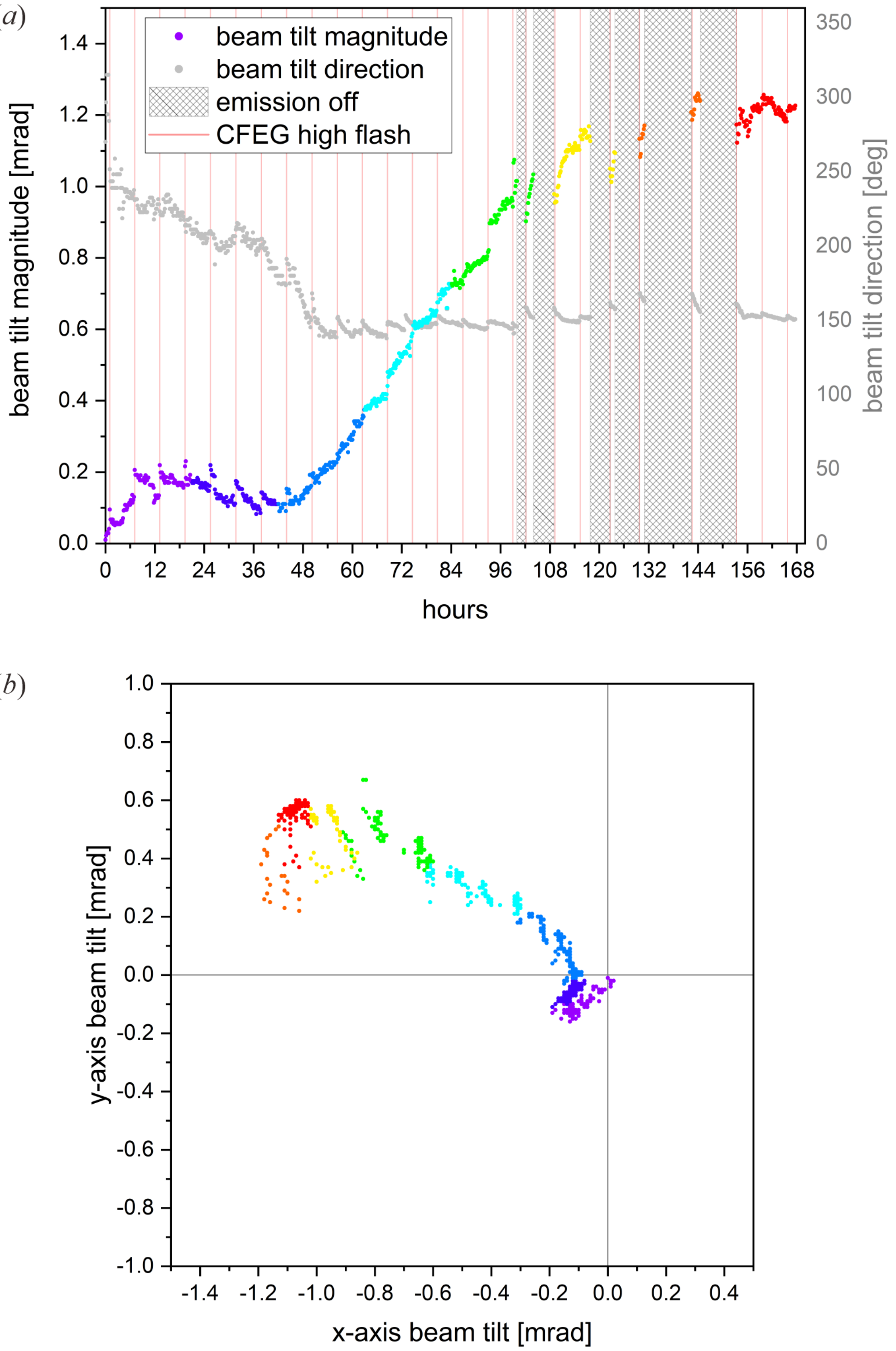

(*b*)

Supplementary Figure S4. Beam tilt stability measurement over the period of a week. (*a*) Beam tilt magnitude and direction versus time. Pink vertical lines indicate CFEG flash points (every 6 hours). Shaded gray areas were periods in which the emission was switched off. (*b*) Cartesian plot of the beam tilt. The points use the same time-based color coding as in (*a*).
